# Evolutionary trends of plague research from 2016 to 2025: A bibliometric analysis

**DOI:** 10.1371/journal.pntd.0014337

**Published:** 2026-05-13

**Authors:** Yanfen Niu, Yihui Zhang, Song Zhou, Guang Liu, Yongming Chen, Yuming Yuan, Guoyi Du, Jue Liu

**Affiliations:** 1 Department of Epidemiology and Biostatistics, School of Public Health, Peking University, Beijing, China; 2 Department of Laboratory, Anti-plague Institute of Hebei Province, Zhangjiakou, Hebei, China; 3 Office, Anti-plague Institute of Hebei Province, Zhangjiakou, Hebei, China‌‌; 4 Key Laboratory of Epidemiology of Major Diseases of the Ministry of Education, Peking University, Beijing, China; 5 Institute for Global Health and Development, Peking University, Beijing, China; University of Texas Medical Branch, UNITED STATES OF AMERICA

## Abstract

**Background:**

Plague is a severe zoonotic disease caused by *Yersinia pestis*, a pathogen characterized by high infectivity and mortality rates. Historically, three global pandemics have inflicted heavy disasters on human society. Despite improvements in control measures and the application of antibiotics, plague has been somewhat controlled; however, since the beginning of the 21st century, plague outbreaks have continued to occur in regions with a high burden of neglected tropical diseases (NTDs) in Africa, the Americas, and Asia. In recent years, the rapid development of technologies such as molecular biology, immunology, and bioinformatics has propelled significant advancements in plague diagnostics, vaccine development, and transmission mechanisms. However, there has been a lack of systematic quantitative analysis of the distribution characteristics, evolving hotspots, and frontier trends of plague research, which makes it challenging to provide comprehensive scientific support for research and control decision-making.

**Methods:**

Search for relevant literatures on plague that were published in the Web of Science Core Collection and PubMed database from January 1, 2016 to November 12, 2025.Bibliometric methods were adopted, and software including COOC 20.6, VOSviewer 1.6.20, and Anaconda were used to analyze the publication trend, distribution of institutions, national cooperation network, keyword co-occurrence clustering, and the annual variation trends.

**Results:**

A total of 1994 documents were finally included. The annual number of publications showed an overall fluctuating upward trend, with a significant growth rate from 2020 to 2021 (annual growth rate of 10.44%). Core research institutions included the U. S. Centers for Disease Control and Prevention and Beijing Institute of Microbiology and Epidemiology. The United States, China, France and Madagascar were the main core countries for cooperation. Keyword co-occurrence clustering identified five major research fields, which were plague vaccine development and immune mechanism, ecology and vector control, historical epidemiology and public health, epidemiology and transmission chain, and plague-related infectious diseases and biosafety. The research trends analysis showed that from 2016 to 2020, the plague research mainly focused on keywords such as “Rodents” “Epidemiological Survey” “Human Plague” and “Fleas”. From 2021 to 2025, “Phylogenetic Analysis,” “Public Health,” and “Madagascar” newly entered the top 20 keyword list; the frequencies of “Black Death” and “Infectious Disease” increased significantly, while the frequencies of “Plague Vaccine” and “Prairie Dogs” remained relatively stable.

**Conclusions:**

Over the past decade, remarkable achievements have been made in plague research. Interdisciplinary integration and technological innovation have continued to deepen. However, global collaboration remains insufficiently developed. In the future, it is necessary to foster broader cross-regional cooperation, accelerate the research, development and translation of vaccines and diagnostic technologies, integrate multiple technologies to construct a precise prevention and control system. and enhance the global collaborative prevention and control capabilities of plague.

## Author summary

Plague is a severe infectious disease caused by *Yersinia pestis*. Historically, plague has caused three global pandemics and continues to reemerge sporadically in regions with a high burden of neglected tropical diseases in Africa, the Americas, and Asia, posing a persistent threat to global public health security. In the past decade, advancements in multidisciplinary technologies such as molecular biology and ecology have led to significant breakthroughs in plague research, including diagnostic techniques, vaccine development, and the study of transmission mechanisms. However, there has been a lack of systematic quantitative analysis regarding the overall distribution of global research, the evolution of hotspots, and frontier trends. This study has employed bibliometric methods to analyze 1,994 plague-related documents indexed in the Web of Science Core Collection and PubMed databases from 2016 to 2025. It has systematically revealed publication trends, institution distribution, national collaboration networks, keyword co-occurrence clustering, and the evolution of hotspots in this field. The research has identified five core research areas: vaccine development and immune mechanisms, ecology and vector control, historical epidemiology and public health, epidemiology and transmission chains, and related infectious diseases and biosafety research. Furthermore, it has found that recent research focuses on vaccine development, molecular tracing, the translation of public health policies, precise control of specific epidemic sources, and integrated prevention of related diseases. The results of this study can provide data support for researchers to clarify research directions and for control departments to optimize decision-making. Additionally, this outcome serves as a reference for strengthening global cross-regional cooperation and constructing a precise prevention and control system, thereby enhancing global collaborative capacity for plague prevention and control.

## Introduction

Plague is a severe infectious disease caused by *Yersinia pestis*. Its highly infectious nature, rapid transmission speed, and high mortality rate continuously pose a threat to global public health security. Throughout human history, there have been three major pandemics of plague, which brought significant disasters to human society [1]. With improved of prevention and control measures and the discovery of streptomycin, plague has been effectively controlled worldwide. However, in the 21st century, plague outbreaks still occur in high-burden regions of neglected tropical diseases in Africa, the Americas, and Asia [2].

Plague is a sylvatic zoonotic disease maintained primarily in rodent reservoirs. Although certain *Yersinia pestis* strains exhibit host-specific adaptations, the long-term persistence of plague in nature depends on a multi-host reservoir complex comprising diverse small mammal species [3]. This multi-host structure parallels the reservoir maintenance mechanisms of rodent-borne neglected tropical diseases such as leishmaniasis [4]. Field investigations have confirmed that in plague-endemic foci of Tanzania, *Rattus rattus* and *Mastomys natalensis* can concurrently harbor *Yersinia pestis* and *Trypanosoma brucei* [5]. Similarly, in Southeast Asia, *Rattus norvegicus* has been found to carry *Leptospira* spp [6]. These findings indicate that certain rodent species may serve as common reservoirs for both plague and various NTDs.

In terms of transmission, plague reaches humans primarily via infected flea bites, following a rodent–vector–human transmission chain. This model closely resembles that of vector-borne NTDs such as leishmaniasis (sand fly) [7] and Chagas disease (triatomine) [8].

In terms of geographical distribution, the natural foci of plague overlap with the endemic areas of various NTDs. In Latin America, plague co-exists with cysticercosis, fascioliasis, and other infectious diseases in certain regions [9]. Sub-Saharan Africa and Madagascar account for over 95% of global human plague cases, while also facing the combined burden of multiple NTDs such as African trypanosomiasis, lymphatic filariasis, and schistosomiasis, resulting in a typical co-endemic pattern [10–12]. Additionally, plague can spread across continents through historical trade and shipping networks, establishing sylvatic cycles in newly invaded areas via local wildlife, thus completing the transition from exotic introduction to long-term local establishment. This pattern of geographical expansion and ecological adaptation parallels the cross-regional transmission and localized epidemic characteristics observed in certain NTDs [13–16]. The overlap in hosts, similarities in vector ecology, and regional disease co-occurrence collectively heighten the resurgence risk of plague in areas with high NTD burdens. These shared ecological characteristics also provide a basis for incorporating plague into a broader NTD prevention and control agenda.

In recent years, with the rapid development of molecular biology, immunology, bioinformatics, and ecology, there have been significant advancements in plague research in areas such as diagnostic technology, vaccine development, and the refinement of prevention and control strategies. The application of molecular detection technologies such as multiplex PCR [17], digital PCR [18], and recombinant enzyme polymerase amplification [19] has significantly improved the accuracy and timeliness of early plague identification; novel antigen screening [20,21] and exploration of immune response mechanisms [22,23] have propelled the development and application of plague vaccines; whole-genome sequencing and bioinformatics analysis have revealed the evolutionary trajectory and adaptive mutation characteristics of *Yersinia pestis* [24,25], providing a molecular basis for targeted interventions; factors such as ecological changes caused by global climate change and human activities disturbing natural epidemic foci have also continuously expanded the direction of plague research [26,27].

Despite significant achievements in existing research, there has been little systematic and quantitative analysis of the global distribution, evolution of hotspots, and frontier trends in plague research. Bibliometric analysis is an important method for quantitatively assessing the development dynamics of a discipline. By mining vast amounts of literature data, it can systematically outline research hotspots, core contributors, and development trends, providing scientific references for researchers to refine their research directions and summarize evidence-based prevention and control data, thereby optimizing decision-making for plague control departments. This method has been widely applied in fields such as infectious diseases and public health [28,29]. Based on this, this study focuses on literature related to plague from 2016 to 2025, employing bibliometric methods to systematically analyze the hotspots and evolution patterns of plague research over the past decade from dimensions such as publication trends, institution distribution, national collaboration networks, and keyword co-occurrence.

This study selects the period from 2016 to 2025 for analysis based on three main considerations. Firstly, this time window comprehensively covers the latest developments in plague research and clearly reflects the impact of the COVID-19 pandemic on the shift in global infectious disease research focus. Secondly, the ten-year span provides a sufficient sample size of literature, which not only enables reliable research trends but also accommodates the publication lag effect of significant plague events. This ensures the scientific validity and accuracy of the analysis results. Thirdly, the 2017 outbreak of urban pneumonic plague in Madagascar was widely regarded by the academic community as a “turning point” in human plague epidemiology [30]. This event has prompted the international community to refocus on this ancient infectious disease, significantly elevated its research priority and further promoted global collaboration in plague research. The selected timeframe encompasses this critical juncture and the subsequent research developments.

## Materials and methods

### Data sources

Data was collected from the Web of Science (WOS) core collection and PubMed database for literature related to *Yersinia pestis* or plague research from January 1, 2016, to November 12, 2025. The WOS core database utilized the search query “*Yersinia pestis*” (TS) OR “plague” (TS) for literature retrieval, while the PubMed database employed the free terms “plague” OR “*Yersinia pestis*” for literature search. The types of literature included were articles and reviews, excluding books, editorials, conference abstracts, letters, news, interviews, errata, and other document types.

### Data processing

All literature retrieved from the two databases was exported in RefWorks or TXT format. The literature data was merged and converted to XLSX format using Co-Occurrence 20.6 (COOC 20.6) software [31]. A combination of software deduplication and manual verification was employed to remove duplicate literature. By reviewing the titles and abstracts of the literature, and consulting the full texts, when necessary, irrelevant literature not related to plague research was excluded. Ultimately, the literature included for analysis was determined.

### Quality control

A quality control strategy combining dual collaboration and standardized processes was adopted to ensure data accuracy and analysis reliability. ①Literature deduplication and screening: After deduplication using software, two researchers independently conducted manual secondary deduplication and thematic relevance screening. For literature with discrepancies, the full texts were reviewed and verified one by one through joint consultation. If necessary, experts in the field of plague prevention and control were consulted to reach a consensus. ②Keyword Standardization: A keyword reference list was established in advance; synonymous, near-synonymous, and abbreviated terms were standardized, such as unifying “Y. pestis” “*Yersinia pestis*” and “Yersinia pestis” to “*Yersinia pestis*” and standardizing “rF1-V antigen” and “recombinant F1-V antigen” to “recombinant F1-V antigen”. Generic terms like “study” and “research” which lack substantive meaning were eliminated.

### Data statistics and visualization analysis

The data was organized using Excel 2020 software, with frequency and percentage used to represent the count data. COOC 20.6 software was employed to extract basic information such as publication year, title, keywords, institutions, and countries or regions from the literature, followed by data cleaning to remove irrelevant literature related to the plague, deduplication, and synonym merging. Anaconda software was utilized to load data packages such as pandas, NumPy, Matplotlib, and scikit-learn for data statistics, and to create visual charts including time trend graphs, collaboration network diagrams, and annual keyword variation charts. VOS viewer 1.6.20 software was used for co-occurrence clustering analysis of keywords.

## Results

### General information

**Fig 1:**
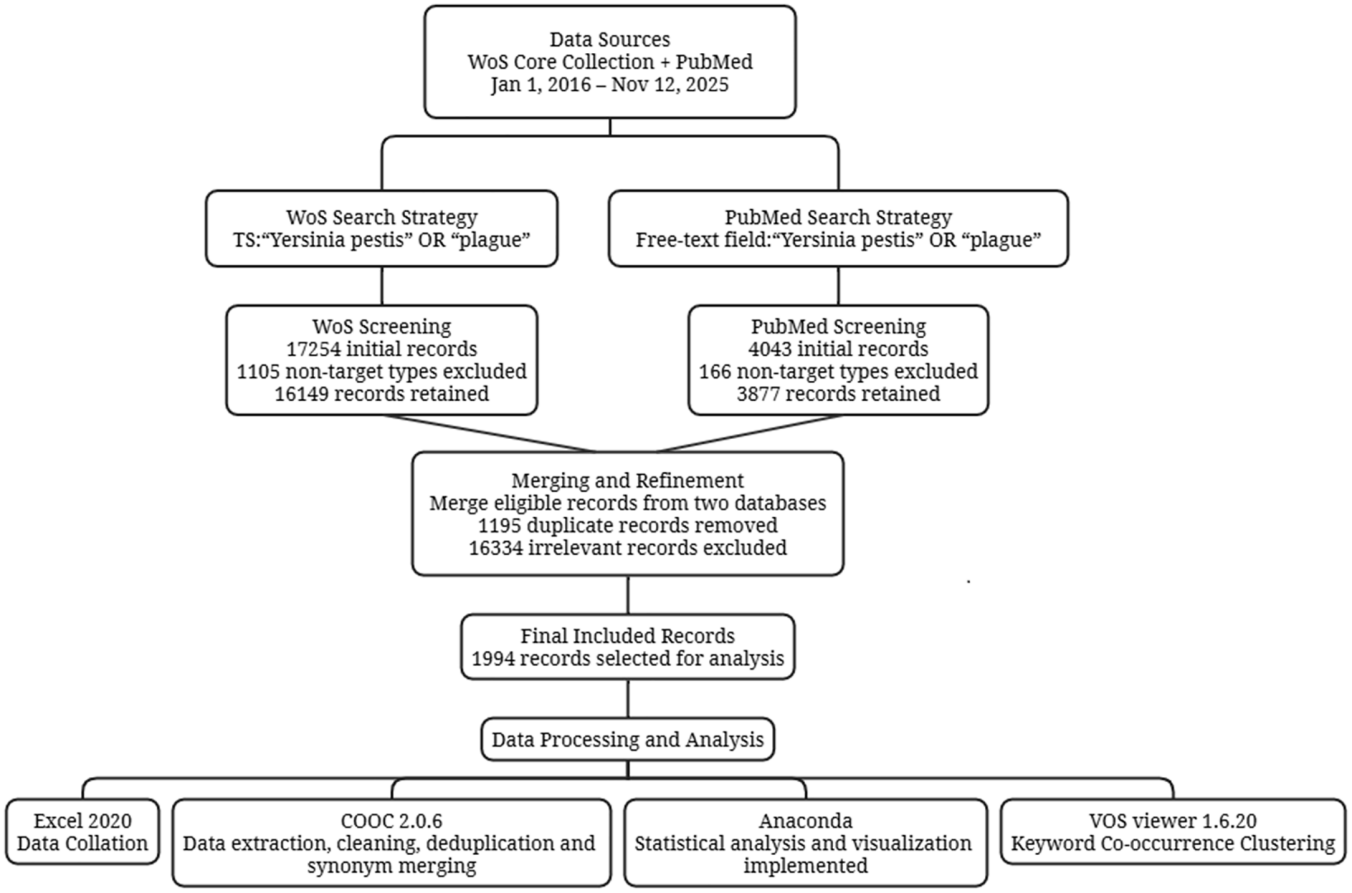
Retrieval and analysis process of plague research literatures (2016-2025).

According to the retrieval and screening strategy of this study, a total of 17,254 plague-related articles were retrieved from the WOS core database from January 1, 2016, to November 12, 2025. After excluding 1,108 non-target document types, 16,146 articles were included. During the same period, an initial search in the PubMed database yielded 4,043 plague-related articles, from which 166 non-target document types were excluded, resulting in 3,877 articles included. After merging the literature from both databases, two researchers conducted a manual secondary deduplication and thematic relevance screening according to standardized procedures, ultimately excluding 1,195 duplicate articles and 16,834 irrelevant articles, confirming 1,994 articles for analysis (Fig 1).

### Publishing trend

**Fig 2:**
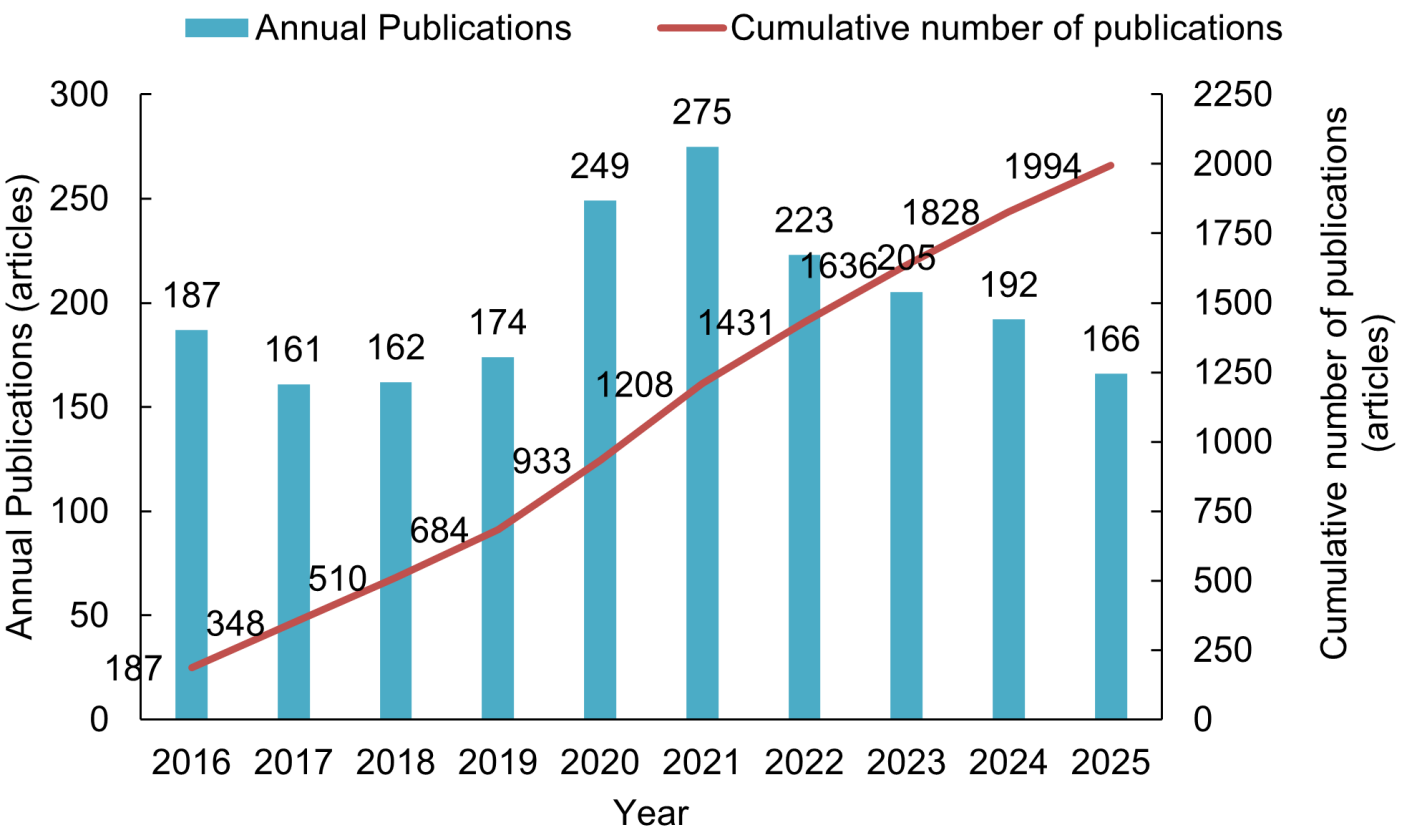
Annual and cumulative number of plague research publications (2016-2025).

The research output on plague from 2016 to 2025 had shown a fluctuating trend. From 2016 to 2019, the fluctuations were relatively minor, with the annual publication volume maintained between 161 and 187 articles, resulting in an average annual output of 171 articles. The period from 2020 to 2021 marked a phase of rapid increase, with the annual publication volume rising from 249 articles to 275 articles, reflecting an annual growth rate of 10.44%. From 2022 to 2025, the publication volume gradually declined, decreasing from 223 articles to 166 articles; however, the average annual output (197 articles) remained higher than that of the 2016-2019 period. Over the past decade, a total of 1,994 articles had been published in plague research, with cumulative proportions for each phase being 34.30%, 26.28%, and 39.42%, indicating a relatively balanced distribution (Fig 2).

### Institution

The 1,994 articles included in this study were affiliated with 2,339 research institutions, with the top 20 institutions collectively publishing 598 papers, representing 30.01% of the total literature. The results showed that the U. S. Centers for Disease Control and Prevention had the highest intermediary centrality (0.064) and ranks third in publication volume (68 articles), while the Beijing Institute of Microbiology and Epidemiology had the highest publication volume (86 articles), with the third highest intermediary centrality at 0.047(Table 1).

**Table 1:** Top 20 institutions by the number of plague research publications (2016-2025).

Institution	Count	centrality
Beijing Institute of Microbiology and Epidemiology	86	0.047
Pasteur Institute of Madagascar	70	0.028
U. S. Centers for Disease Control and Prevention	68	0.064
United States Geological Survey	55	0.013
Russian Academy of Sciences	50	0.027
Chinese Center for Disease Control and Prevention	41	0.038
Pasteur Institute	40	0.052
Colorado State University	37	0.016
Israel Institute for Biological Research	34	0.003
University of Oslo	32	0.044
University of Cambridge	31	0.041
National Institute of Allergy and Infectious Diseases	31	0.040
University of Texas Medical Branch	29	0.023
University of Antananarivo	29	0.011
Aix-Marseille University	27	0.025
Tsinghua University	26	0.038
State Research Center for Applied Microbiology and Biotechnology	24	0.007
University of Oxford	23	0.037
University of Lille	23	0.014
Chinese Academy of Sciences	22	0.032

### Cooperation among countries in publishing

**Fig 3:**
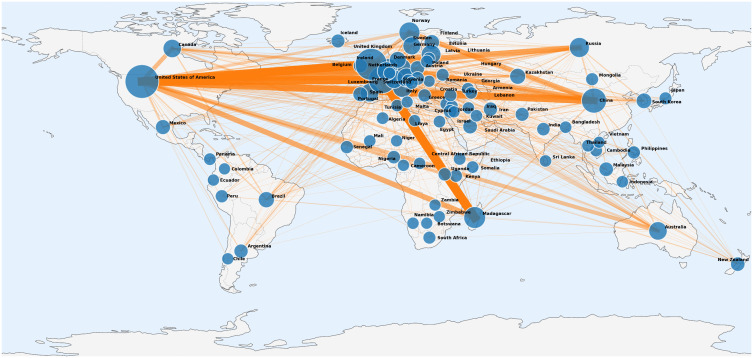
Research cooperation network diagram of countries publishing on plague (2016-2025). The size of the dots reflects the number of publications, and the width of the connecting lines indicates the degree of cooperation among countries. Base map country borders and coastlines are from Natural Earth (public domain, https://www.naturalearthdata.com/about/terms-of-use/).

A total of 110 countries was involved in the literature, with 468 collaborative papers accounting for 23.47% of the total literature. The top ten countries ranked by collaboration frequency were the United States, China, France, the United Kingdom, Germany, Madagascar, Canada, Norway, Russia, and Australia. The collaboration frequency among the top ten countries accounted for 30.50% of the total collaboration frequency (510/1672). In bilateral cooperation, countries with a collaboration frequency greater than 30 times included France and Madagascar (33 times), the United States and the United Kingdom (32 times), and France and the United States (31 times). Countries with collaboration frequencies ranging from 20 to 30 times included the United Kingdom and Madagascar (29 times), China and the United States (27 times), Germany and the United States (26 times), France and the United Kingdom (25 times), Germany and the United Kingdom (22 times), the United States and Russia (21 times), and Madagascar and the United States (20 times). Fig 3 illustrates the collaborative network of countries publishing research on plague from 2016 to 2025.

### Keyword co-occurrence cluster analysis

**Fig 4:**
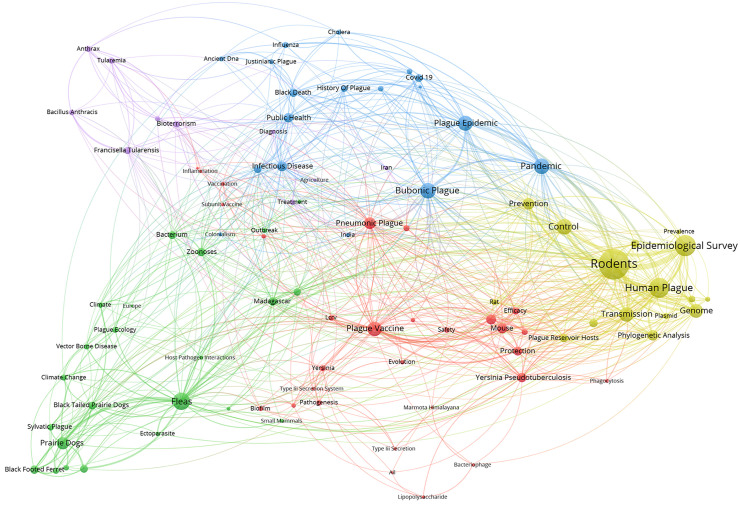
Keyword co-occurrence clustering diagram of plague research (2016-2025). Red represents cluster 1, green represents cluster 2, blue represents cluster 3, yellow represents cluster 4, purple represents cluster 5. The size of nodes indicates the frequency of keywords, and the thickness of connecting lines represents the strength of keyword co-occurrence.

A total of 5,067 keywords were extracted from the literature. Among them, 97 high-frequency keywords appeared at 8 times or more. After excluding the search terms “plague” and “*Yersinia pestis*” co-occurrence cluster analysis was conducted on the remaining high-frequency keywords, ultimately forming five core research clusters (Fig 4). Cluster 1 (Red) focuses on the research themes of plague vaccine development and immune mechanisms, with core keywords including “Plague Vaccine” “Pneumonic Plague” “Mouse” and “F1 Antigen”. Cluster 2 (Green) centers on the ecological environment of plague and vector control, with core keywords such as “Fleas” “Prairie Dogs” “Zoonoses” and “Madagascar”. Cluster 3 (Blue) addresses the historical epidemiology and public health aspects of plague, with core keywords including “Pandemic” “Bubonic Plague” “Plague Epidemic” and “Public Health”. Cluster 4 (Yellow) investigates the epidemiology and transmission chains of plague, with core keywords such as “Rodents” “Epidemiological Survey” “Human Plague “and “Transmission”. Cluster 5 (Purple) explores plague-related infectious diseases and biosafety, with core keywords including “*Francisella Tularensis*” “Bioterrorism” “Anthrax” and “Tularemia”.

### Keyword annual variation analysis

**Fig 5:**
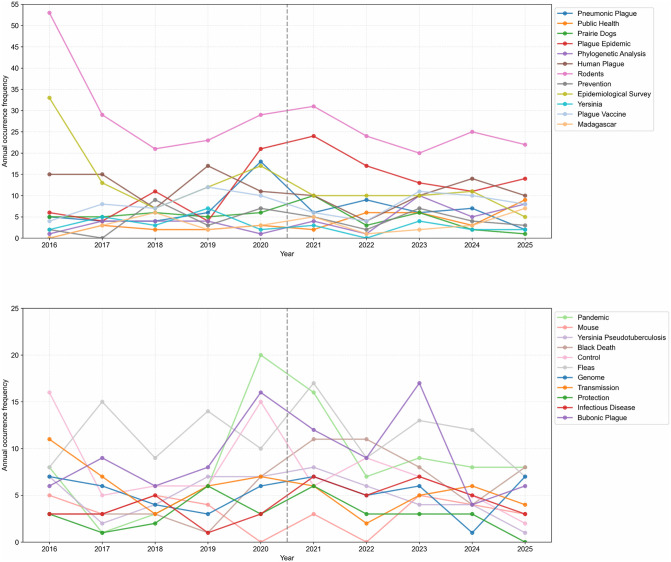
Annual variation trend of keywords in plague research (2016-2025). Among the top 20 keywords, 17 were common to both phases, 3 appeared only in the first phase (“Yersinia” “Mouse” “Protection”), and 3 appeared only in the second phase (“Phylogenetic Analysis” “Public Health” “Madagascar”). To more clearly demonstrate keyword changes over the decade, the 26 keywords are divided into two subfigures.

The research on plague over a decade was divided into two phases based on five-year intervals: 2016–2020 and 2021–2025. After excluding the search terms “plague” and “*Yersinia pestis*”, a comparative analysis of the top 20 keywords by frequency was conducted (Fig 5). The results indicated that the keywords in the first phase predominantly included “Rodents” “Epidemiological Survey” “Human Plague” and “Fleas”, focusing on basic research such as investigations of natural plague foci, studies on hosts and vectors, and disease type research. In the second phase, the keywords that newly entered the top 20 included “Phylogenetic Analysis” “Public Health” and “Madagascar”, while the frequencies of “Black Death” and “Infectious Disease” significantly increased. The terms “Plague Vaccine” and “Prairie Dogs” maintained relatively stable frequencies across both phases.

## Discussion

This study found that the cumulative publication volume of research on plague from 2016 to 2025 was steadily increasing, with annual publication volumes showing stable fluctuations. Notably, the rapid growth in publication volume from 2020 to 2021 may be related to the heightened emphasis on infectious disease research in the global health sector. Particularly in the context of the COVID-19 pandemic, research on the transmission mechanisms of infectious disease pathogens and prevention and control technologies received widespread attention [32,33], indirectly boosting the research interest in traditional severe infectious diseases such as plague. Although there was a decline in publication volume after 2022, the average annual publication volume remained higher than that of 2016–2019. This indicates that plague, as a potential public health threat, continues to receive stable attention in research. The composition ratio of the cumulative publication volume over the decade also reflected this point.

The analysis of the literature on plague research over the past decade revealed that the U. S. Centers for Disease Control and Prevention and the Beijing Institute of Microbiology and Epidemiology both demonstrated high productivity and strong collaborative hub attributes. It is evident that these two institutions played a core coordinating role in global plague research. Future efforts will focus on strengthening deep cooperation between them to promote technology sharing and joint problem-solving.

Research results from the National Cooperative Network indicated that the plague research collaboration network exhibited a phenomenon where core countries such as the United States, China, and France collaborate closely, while global cooperation was concentrated among core countries (with core countries accounting for 30.50% of collaboration frequency). Many countries had limited participation in international collaborations, with multi-country collaborative literature only constituting 23.47%. The study suggested that future efforts should focus on strengthening research collaborations between core networks and non-core countries. In bilateral cooperation, there was close collaboration among countries such as France, Madagascar, the United States, and the United Kingdom, which was closely related to Madagascar being a high-incidence area for plague and the complementary advantages of advanced research technologies possessed by European and American countries [34–36]. This model parallels the North–South cooperative paradigm commonly observed in leishmaniasis research, meriting emulation by regions with concentrated plague foci and endemic NTDs, including Africa and Central Asia [37]. Consequently, plague prevention and control should not proceed in isolation but rather be incorporated into a wider NTD collaborative framework, enhancing integrated response in high-burden regions. As a developing country, China also occupied an important position in the global cooperation network, with 27 collaborations with the United States, ranking high among bilateral cooperation relationships, reflecting China’s international influence in the field of plague research. However, compared with core countries such as the United States and France, China’s multi-directional cooperation remains relatively limited, and collaboration with NTD-high-burden and plague-endemic countries is even scarcer. This indicates that China needs to further strengthen international cooperation to jointly advance in-depth research on plague.

Keyword co-occurrence clustering and annual variation results indicated that research on plague focused on five core areas over the past decade, showing a clear research trend. The development of plague vaccines and the study of immune mechanisms (Cluster 1) was consistently a focal point of research. In recent years, vaccine research centered on subunit vaccines [38] and live attenuated vaccines [39], while also rapidly advancing new vaccine technologies such as nucleic acid vaccines [20]. Additionally, it was confirmed that both cellular immunity and humoral immunity play protective roles in plague infections [21]. Following the COVID-19 pandemic, the global public health emergency response system saw a surge in demand for technologies to prevent and control severe infectious diseases, which propelled the translation of plague vaccines from laboratory research to clinical trials [40,41]. The persistent high frequency of the keyword “Plague Vaccine” from 2016 to 2025 further corroborated the long-term significance of this direction. Future research should focus more on the safety of vaccines, the long-term efficacy of protective effects, and their applicability in field settings, while strengthening the connection between basic research and clinical translation to provide more efficient technological reserves for plague emergency prevention and control.

Cluster 2 focused on plague ecology and vector control research, emphasizing the interactions among fleas, prairie dogs, and other reservoir hosts within their natural ecosystems.. Particularly in concentrated epidemic areas such as Madagascar, the United States, and China, related research holds significant practical value for plague prevention and control [42–44]. Eads, David A. et al. [45] found that fipronil and its metabolites have lethal effects on flea larvae. However, Lemaitre, Nadine et al. [46] discovered that plague strains carrying the multi-drug-resistant plasmid pIP1202 are transmitted among fleas. This indicates that traditional chemical control methods face challenges, necessitating the development of new vector control technologies. The impact of environmental and climate changes on host-vector-pathogen interactions was also a key focal point during the study period, with studies striving to construct ecological predictive models to quantify the effects of climate change on host habitat distribution [27]. The stability of keywords such as “Prairie Dogs” and “Fleas” in annual variations reflected the foundational and critical nature of research in this field. From 2016–2025, the appearance of “Madagascar” among the top 20 keywords was strongly associated with the 2017 pneumonic plague epidemic in Antananarivo [47]. As a country with one of the world’s highest plague burdens and multiple overlapping NTDs, Madagascar’s increasing research focus signals both a demand for precise control in specific foci and a broader shift of research resources toward co-endemic, high-burden regions. Future efforts should enhance interdisciplinary collaboration, develop new vector control technologies, and establish dynamic ecological monitoring networks in these conditions in epidemic areas to improve the foresight and precision of prevention and control. The historical epidemiology and public health research of plague (Cluster 3) combines ancient DNA techniques with historical epidemiological methods to reveal the transmission pathways and evolutionary characteristics of historical pandemics such as the Black Death [48]. Influenced by the COVID-19 pandemic, this field shifted from mere historical verification to providing experiential references for addressing global modern public health crises. Comparative studies [49–51] conducted by multiple international teams on COVID-19 and historical plague prevention and control have summarized significant experiences and lessons learned from major epidemic prevention and control efforts, extracting universal emergency response and risk management strategies for infectious diseases. This provides theoretical support for the collaborative prevention and control of plague and other infectious diseases. The emergence of “Public Health” as a high-frequency keyword in the second phase, alongside the increased research interest in “Infectious Disease”, collectively indicates that plague research has evolved from the prevention of a single disease to the co-prevention of multiple diseases. It also signifies a transition from academic exploration to the application and transformation of public health policies, which is expected to be one of the future research hotspots.

Cluster 4 focused on the epidemiology and transmission chain of plague, clarifying the transmission routes and epidemic characteristics of human plague through molecular epidemiological investigations, thereby providing technical support for rapid epidemic response. Lovasoa Nomena et al. [52] established a new typing standard based on whole-genome sequencing data, which improved the accuracy of strain tracing from traditional regional levels to individual levels. Keener, Rachel M et al. [53] discovered the association between single nucleotide polymorphisms in the FCRL3 gene and susceptibility to plague in populations through genome-wide association studies, providing new technical support for precise prevention and control of plague. The annual variations in keywords such as “Rodents”, “Epidemiological Survey”, “Human Plague”, and “Phylogenetic Analysis” also indicated that this field gradually evolved from traditional epidemiological investigations and basic research on transmission chains to molecular tracing and precise prevention and control.

Research on plague-related infectious diseases and biosafety falls under Cluster 5, which focused on zoonotic diseases such as bioterrorism and anthrax. This reflected the trend of collaborative development between plague research and other fields. Due to the high pathogenicity of *Yersinia pestis* and its potential for misuse, biosafety concerns have always been a focal point. The research direction gradually expanded from early monitoring of a single pathogen to joint monitoring [54], joint diagnosis [55], and joint vaccine [56] development of plague alongside anthrax and tularemia, forming a research model for the coordinated prevention and control of multiple pathogens. This trend not only responded to the needs of the global biosafety system construction but also provided technical support for comprehensive responses to emerging infectious diseases.

This study had certain limitations. Firstly, due to language constraints, the literature included in this research was limited to the internationally recognized WOS core collection and PubMed database, excluding non-English international literature. This may have resulted in the omission of non-English publications, leading to an underestimation of plague research in certain countries. Secondly, metrics such as citation frequency and research quality evaluation were not included, which cannot comprehensively reflect the impact of the research. Future studies should expand the range of literature sources and incorporate literature quality evaluation indicators to further enhance the accuracy and comprehensiveness of the analysis results.

In summary, significant progress was made in research on plague over the past decade; however, numerous challenges remain. In the future, we should accelerate the development and promotion of novel vaccines and rapid diagnostic reagents, while strengthening research on immune mechanisms and resistance mechanisms. Enhancing interdisciplinary and cross-regional cooperation is essential, as well as deepening the collaborative model of field investigation-laboratory research-policy translation, expanding the international cooperation network, and improving the overall level of global plague prevention and control. Additionally, integrating technologies such as artificial intelligence and big data to establish a dynamic monitoring and epidemic early warning system for plague and other severe infectious diseases will provide scientific support for their coordinated prevention and control.

## Supporting information

S1 Table Top 20 institutions by the number of plague research publications (2016–2025). (XLSX)

S1 Fig Retrieval and analysis process of plague research literatures (2016–2025). (TIF)

S2 Fig Annual and cumulative number of plague research publications (2016–2025). (TIF)

S3 Fig Research cooperation network diagram of countries publishing on plague (2016–2025). The size of the dots reflects the number of publications, and the width of the connecting lines indicates the degree of cooperation among countries. Base map country borders and coastlines are from Natural Earth (public domain, https://www.naturalearthdata.com/about/terms-of-use/).
(TIF)

S4 Fig Keyword co-occurrence clustering diagram of plague research (2016–2025). Red represents cluster 1, green represents cluster 2, blue represents cluster 3, yellow represents cluster 4, purple represents cluster 5. The size of nodes indicates the frequency of keywords, and the thickness of connecting lines represents the strength of keyword co-occurrence.
(TIF)

S5 Fig Annual variation trend of keywords in plague research (2016–2025). Among the top 20 keywords, 17 were common to both phases, 3 appeared only in the first phase (“Yersinia” “Mouse” “Protection”), and 3 appeared only in the second phase (“Phylogenetic Analysis” “Public Health” “Madagascar”). To more clearly demonstrate keyword changes over the decade, the 26 keywords are divided into two subfigures.
(TIF)

S1 Data PubMed literature search results for plague (2016–2015). (TXT)

S2 Data WoS literature search results for plague (2016–2015). (TXT)

S3 Data Analysis data of plague literature (2016–2015). (XLSX)
