# Supplementary figures and images for: Evolutionary trends of plague research from 2016 to 2025: A bibliometric analysis
Source: PLoS Negl Trop Dis. 2026 May 13;20(5):e0014337. doi: 10.1371/journal.pntd.0014337 (PMC13186357; doi:10.1371/journal.pntd.0014337)

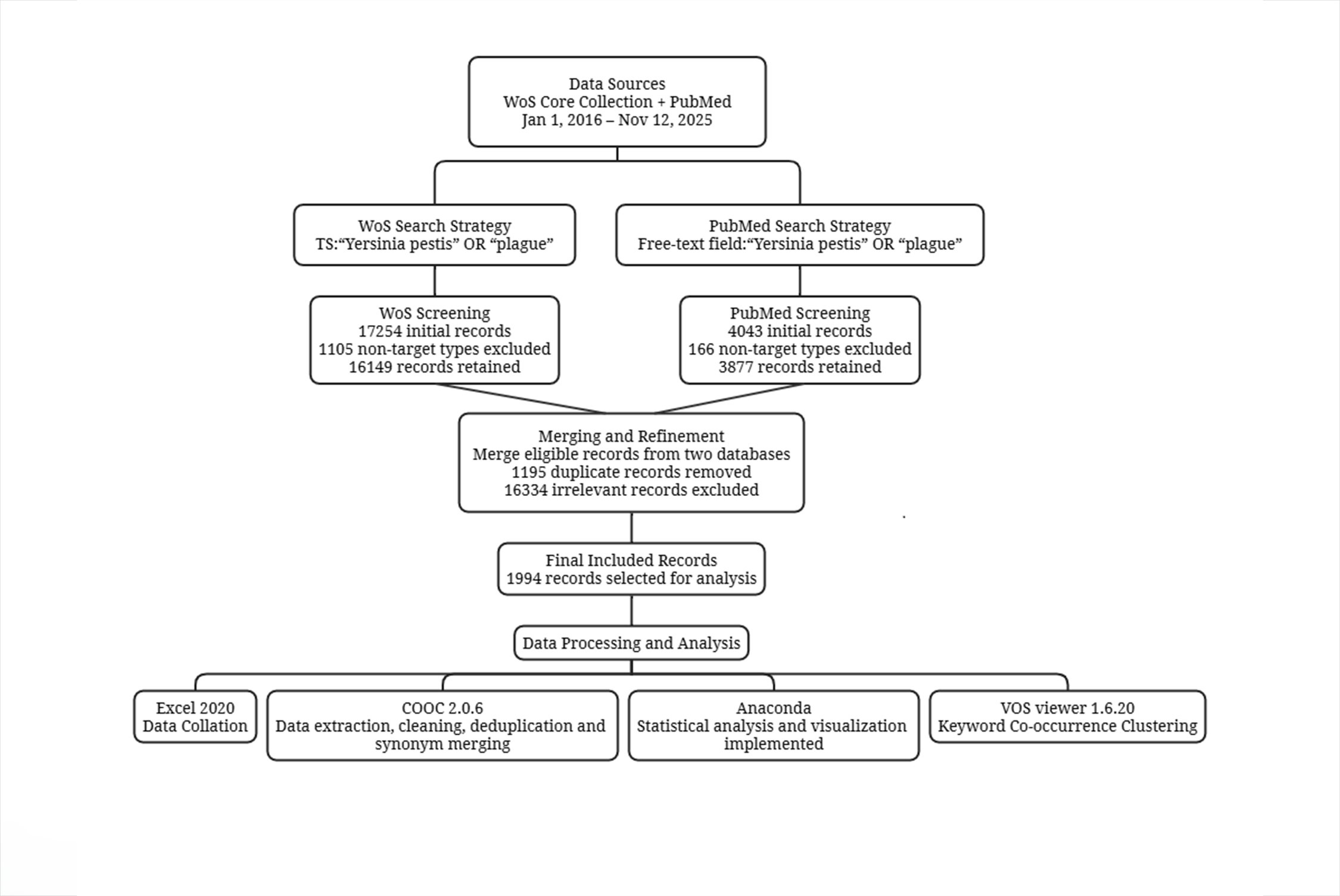

Supplement: S1 Fig — (TIF) [file pntd.0014337.s002.tif]

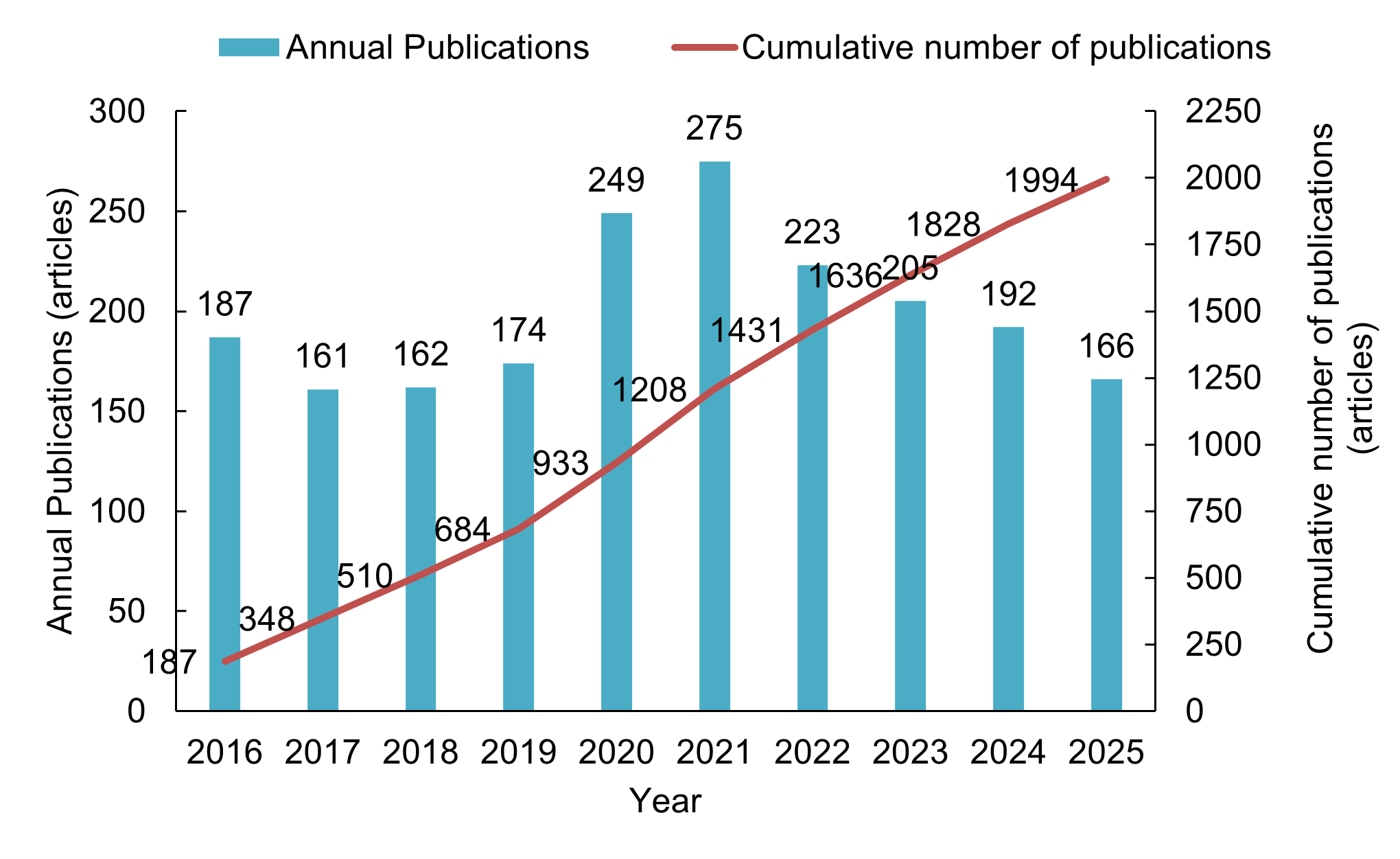

Supplement: S2 Fig — (TIF) [file pntd.0014337.s003.tif]

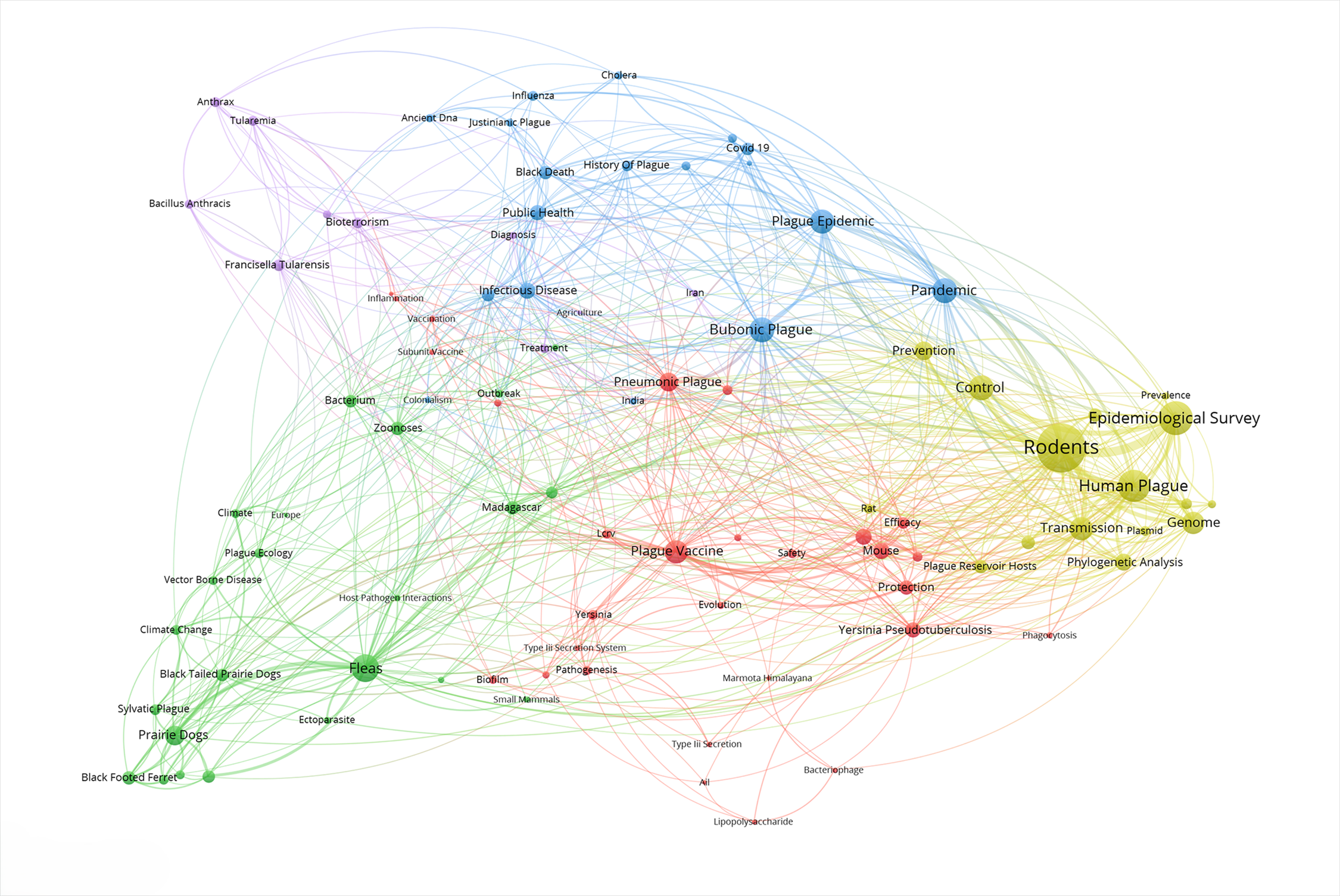

Supplement: S4 Fig — Red represents cluster 1, green represents cluster 2, blue represents cluster 3, yellow represents cluster 4, purple represents cluster 5. The size of nodes indicates the frequency of keywords, and the thickness of connecting lines represents the strength of keyword co-occurrence. (TIF) [file pntd.0014337.s005.tif]
